# Supplementary material for: Direct cortical feedforward and feedback pathways differentially shape visual responses and selectively engage looped circuits
Source: bioRxiv. 2026 Oct 30:2026.10.27.762146. Preprint. [Version 1] doi: 10.64898/2026.10.27.762146 (PMC13733965; doi:10.64898/2026.10.27.762146)
Supplement: Supplementary file 1 [file NIHPP2026.10.27.762146V1-supplement-1.pdf]

bioRxiv preprint doi: https://doi.org/10.64898/2026.10.27.762146; this version posted October 30, 2026. The copyright holder for this preprint (which was not certified by peer review) is the author/funder, who has granted bioRxiv a license to display the preprint in perpetuity. It is made available under a CC-BY 4.0 International license.

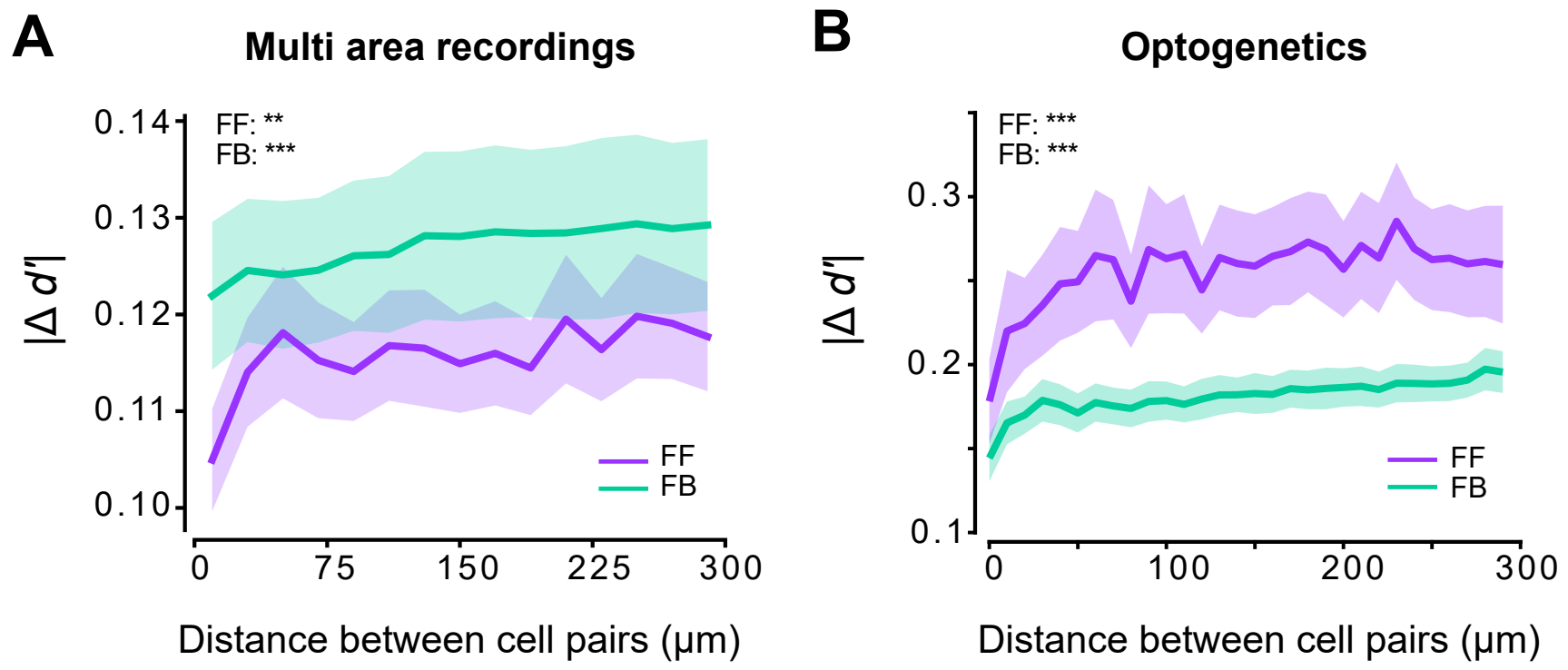

Figure S1. Neighbouring neurons are influenced by FF and FB more similarly.

A) Quantification of the absolute difference in modulation between pairs of neurons as a function of the distance between them in the multi area recordings. Both high FF and high FB induce more similar modulation in neurons that are in close proximity (one-way repeated measures ANOVA per pathway, FF: F=60.9, p=1.28 x 10^{-12}, FB: F=6.7, p=0.010. Line and shading indicate mean and s.e.m. across sessions (n=12 from N=8 mice).

B) Same as a. for optogenetics experiments. Friedman test per pathway, FF: $\chi(29)$=114.6, p=3.87 x 10^{-12}, FB: $\chi(29)$=180.2, p=9.09 x 10^{-24}. Line and shading indicate mean and s.e.m. across sessions (FF: n=30 from N=16 mice, FB: n=23 from N=12 mice).

bioRxiv preprint doi: https://doi.org/10.64898/2026.10.27.762146; this version posted October 30, 2026. The copyright holder for this preprint (which was not certified by peer review) is the author/funder, who has granted bioRxiv a license to display the preprint in perpetuity. It is made available under a CC-BY 4.0 International license.

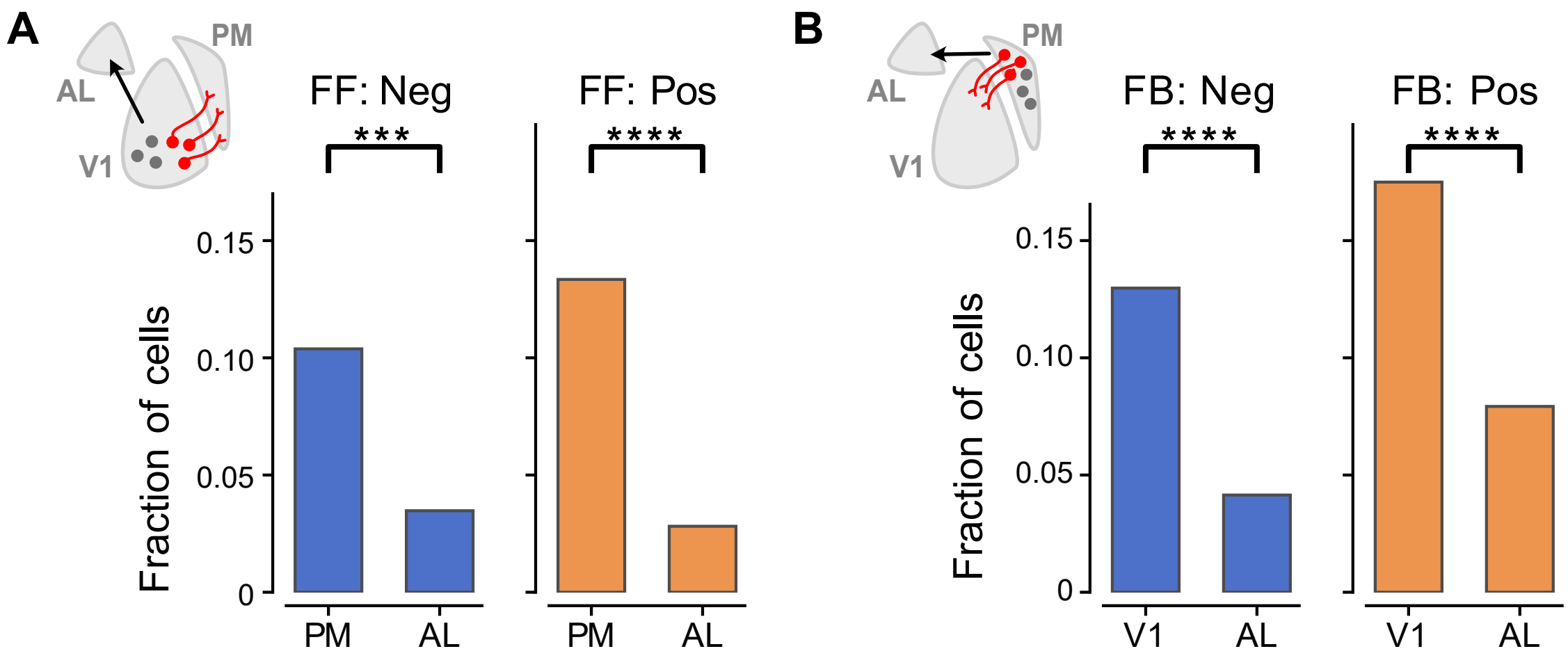

Figure S2: Projection neurons modulate activity in a projection-target specific manner.

A) Fraction of negatively and positively modulated neurons by the FF metric ($V1_{PM}$-$V1_{ND}$) in visual areas AL and PM. A significantly smaller fraction of AL neurons than PM neurons was modulated by the state of the V1 to PM pathway (negative: χ^2 = 14.41, p = 0.00147, positive: χ^2 = 27.55, p = 1.53 x 10^{-7}; n = 5943 PM neurons, N = 8 mice; n = 302 AL neurons, N = 3 mice).
B) The same but testing the specificity of the labeled PM to V1 feedback pathway. The fraction of negatively and positively modulated neurons was lower in AL than in V1 (negative: χ^2 = 27.04, p = 1.99x10^{-7}, positive: χ^2 = 24.76, p = 6.5x10-7; n = 10197 V1 cells, N = 8 mice; n = 410 AL cells, N = 3 mice).

bioRxiv preprint doi: https://doi.org/10.64898/2026.10.27.762146; this version posted October 30, 2026. The copyright holder for this preprint (which was not certified by peer review) is the author/funder, who has granted bioRxiv a license to display the preprint in perpetuity. It is made available under a CC-BY 4.0 International license.

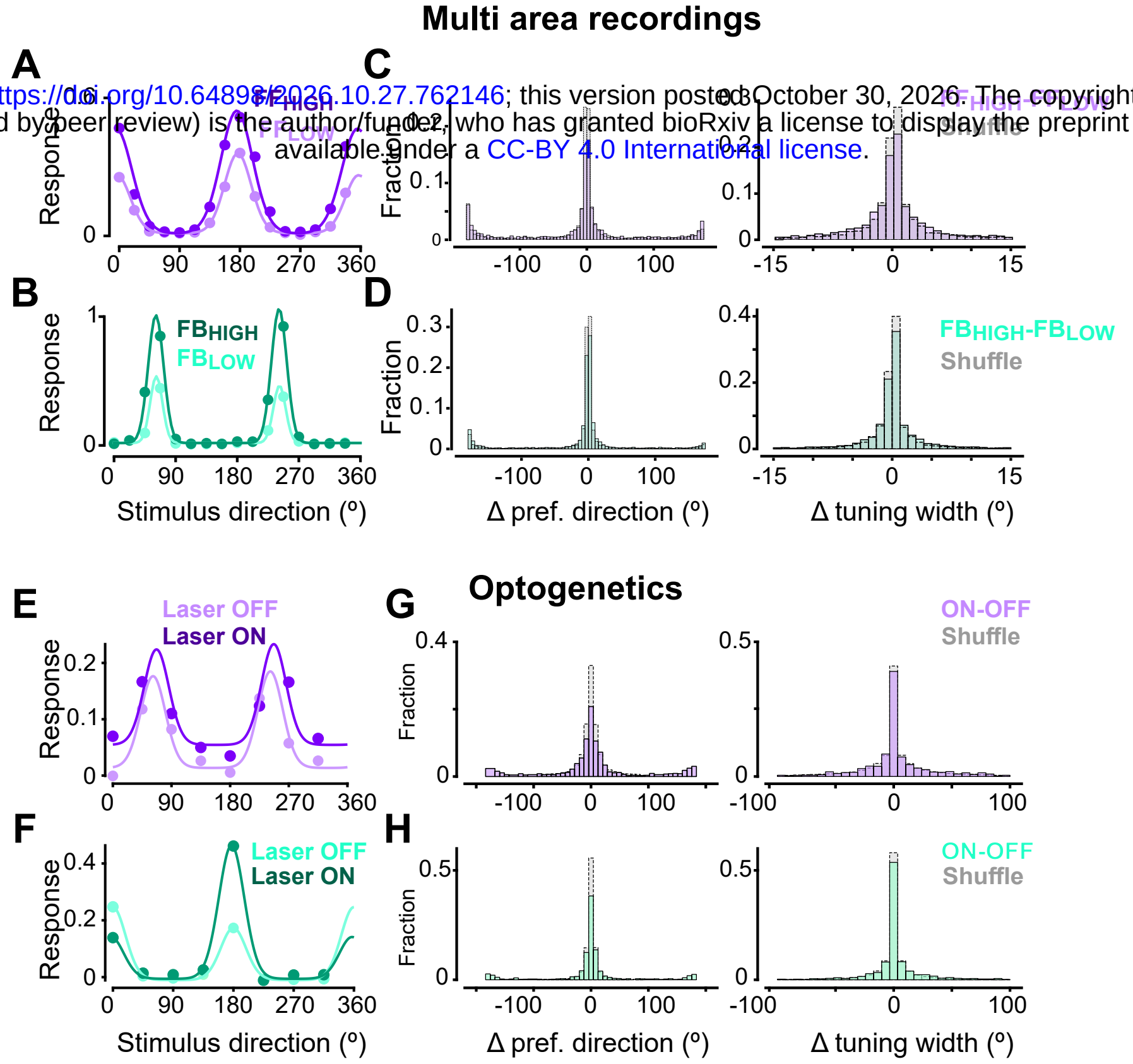

Figure S3. Preferred direction and tuning width remain unchanged across FF and FB HIGH/LOW trials and photostimulation.

A) Example von Mises fit on a PM neuron during FF_{LOW} and FF_{HIGH} trials.
B) Same as A, but for a V1 neuron during FB_{HIGH} and FB_{LOW} trials.
C) Difference in preferred direction (left) and tuning width (right) of PM neurons between FF_{LOW} and FF_{HIGH} trials. Grey indicates shuffle with the difference in parameter values between two fits on random subsets of 25% of trials. The difference in tuning parameters is within the variability observed in random subsets of trials. n = 3016 neurons from N = 8 mice.
D) Same as C, but difference for V1 neurons during FB_{HIGH} and FB_{LOW} trials. n = 5306 neurons from N = 8 mice.
E-H): Same as A-D but for optogenetics experiments. Grey indicates shuffled Laser OFF and Laser ON trials. FF: n =1092 neurons from N=16 mice, FB: n =1381 neurons from N=12 mice.

bioRxiv preprint doi: https://doi.org/10.64898/2026.10.27.762146; this version posted October 30, 2026. The copyright holder for this preprint (which was not certified by peer review) is the author/funder, who has granted bioRxiv a license to display the preprint in perpetuity. It is made available under a CC-BY 4.0 International license.

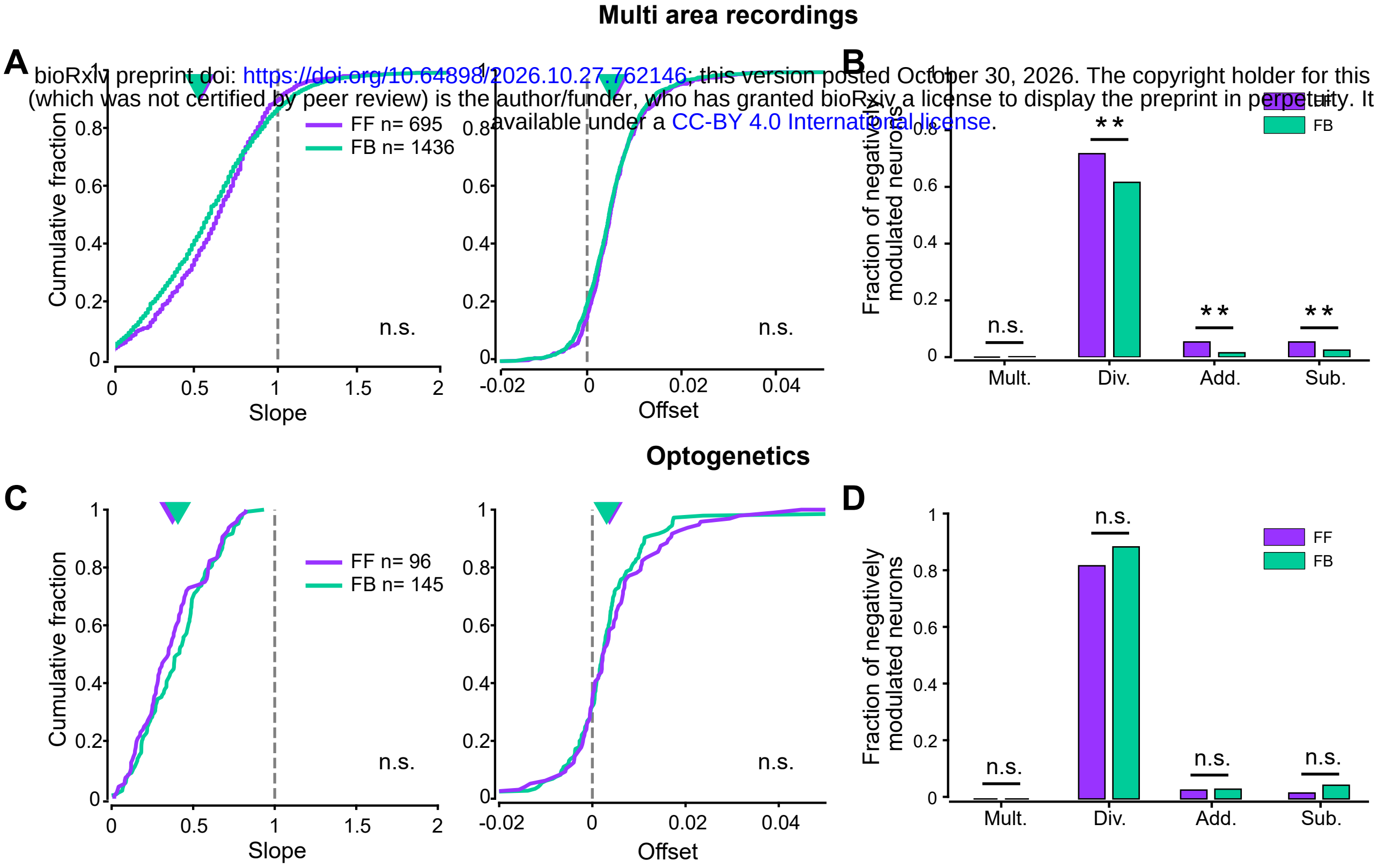

Figure S4. Effect of FF and FB pathways on the visual responses of negatively modulated cells.

A) Cumulative distribution of multiplicative slope (left) and additive offset (right) of cells negatively modulated by high FF/FB activity. Slope: FF: 0.455, FB: 0.461, p=1; Offset: FF: 0.005, FB: 0.004, p=0.0912, Mann-Whitney U test with Bonferroni correction for multiple comparisons. N=8 mice.
B) Fraction of significantly multiplicative, divisive, additive and subtractive neurons. Asterisks denote significance of a chi-square test of proportions (Div. χ = 11.28, p = 0.0063, Add. χ = 12.51, p = 0.0032, Sub. χ = 10.89, p = 0.0077).
C-D): Same as A,B but for FF/FB photostimulation. For each neuron, only stimuli for which the visual response are not completely abolished are used for the fit. Slope: FF: 0.372, FB: 0.406, p=0.18; Offset: FF: 0.003, FB: 0.003, p=0.49, Mann-Whitney U test. FF: N=10 mice, FB: N=10 mice.

bioRxiv preprint doi: https://doi.org/10.64898/2026.10.27.762146; this version posted October 30, 2026. The copyright holder for this preprint (which was not certified by peer review) is the author/funder, who has granted bioRxiv a license to display the preprint in perpetuity. It is made available under a CC-BY 4.0 International license.

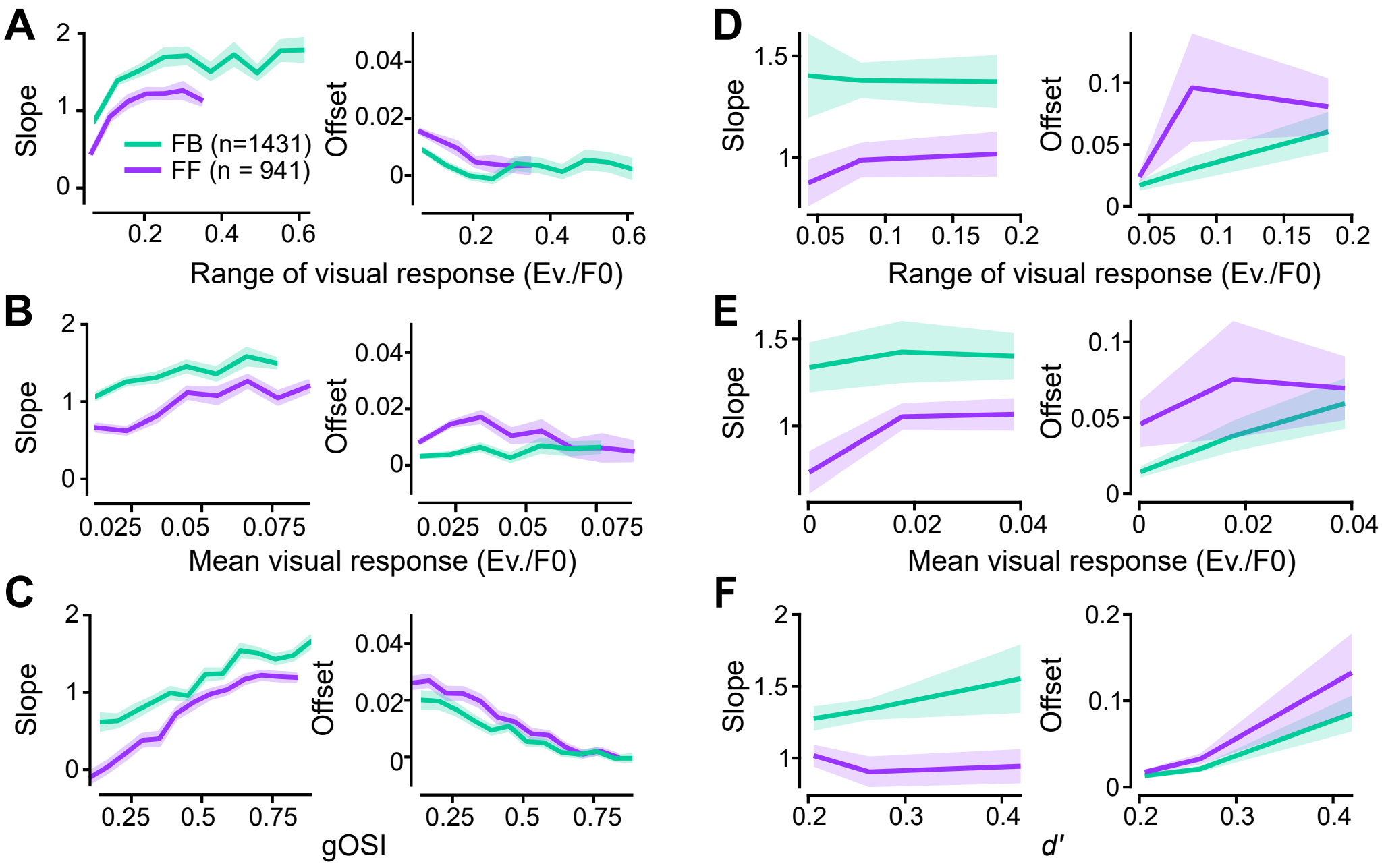

Figure S5. Pathway-specific slope and offset differences do not depend on visual response range and amplitude, orientation selectivity or *d′*.

A) Slope and offset as a function of the range of the visual response across stimulus conditions for multi area recordings. t-statistic and p-value for the pathway effect (ANCOVA). Slope: t = 13.7, p = 1.74 x 10^{-41}. Offset: t = -9.1, p = 1.57 x 10^{-19}. N = 8 mice.
B) Same as A for mean visual response. Slope: t = 15.9, p = 1.51 x 10^{-54}. Offset: t = -10.0, p = 2.75 x 10^{-23}.
C) Same as A for global orientation selectivity index (gOSI). Slope: t = 11.6, p = 1.47 x 10^{-30}. Offset: t = -5.6, p = 2.74 x 10^{-8}.
D) Slope and offset as a function of the range of the visual response across stimulus conditions for optogenetics experiments. Slope: t = -3.3, p =0.001. Offset: t = 2.1, p = 0.038.
E) Same as D for mean visual response. Slope: t = -3.2, p =0.001. Offset: t = 1.8, p = 0.067.
F) Same as D for *d′*. Slope: t = -3.5, p =0.0006. Offset: t = 0.9, p = 0.356.

bioRxiv preprint doi: https://doi.org/10.64898/2026.10.27.762146; this version posted October 30, 2026. The copyright holder for this preprint (which was not certified by peer review) is the author/funder, who has granted bioRxiv a license to display the preprint in perpetuity. It is made available under a CC-BY 4.0 International license.

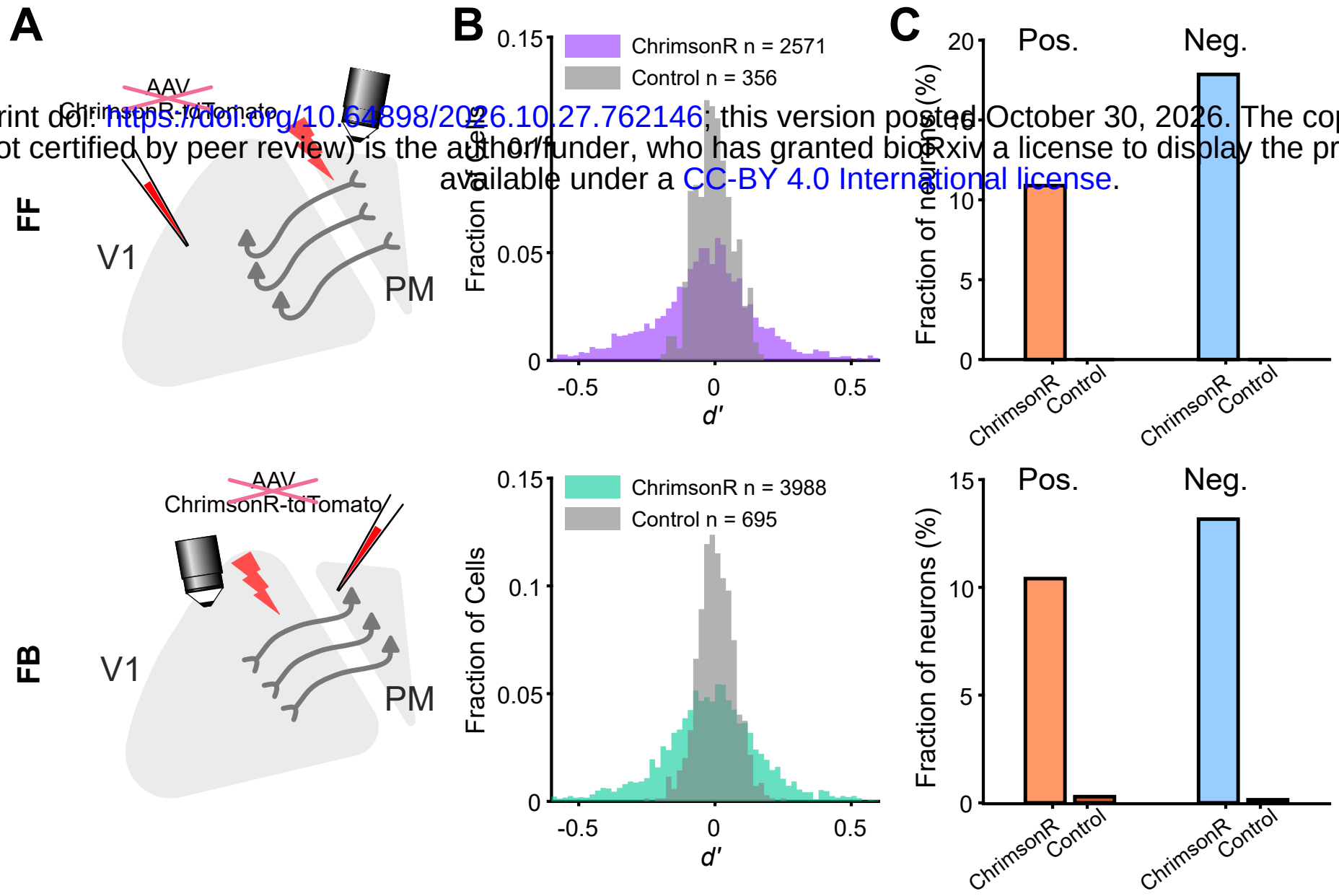

Figure S6: Photostimulation without ChrimsonR expression does not induce visual response modulation.

A) Scheme depicting the experimental paradigm: photostimulation and 2P recording in PM (top) or V1 (bottom) without injection of the ChrimsonR-tdTomato AAV.
B) Distribution of FF (top) and FB (bottom) photostimulation modulation in mice expressing (colored; FF: N=3 mice; FB: N=2 mice) or not expressing (gray) ChrimsonR.
C) Fraction of significantly positively (orange) and negatively (blue) modulated neurons by FF (top) and FB (bottom) photostimulation with and without ChrimsonR.

bioRxiv preprint doi: https://doi.org/10.64898/2026.10.27.762146; this version posted October 30, 2026. The copyright holder for this preprint (which was not certified by peer review) is the author/funder, who has granted bioRxiv a license to display the preprint in perpetuity. It is made available under a CC-BY 4.0 International license.

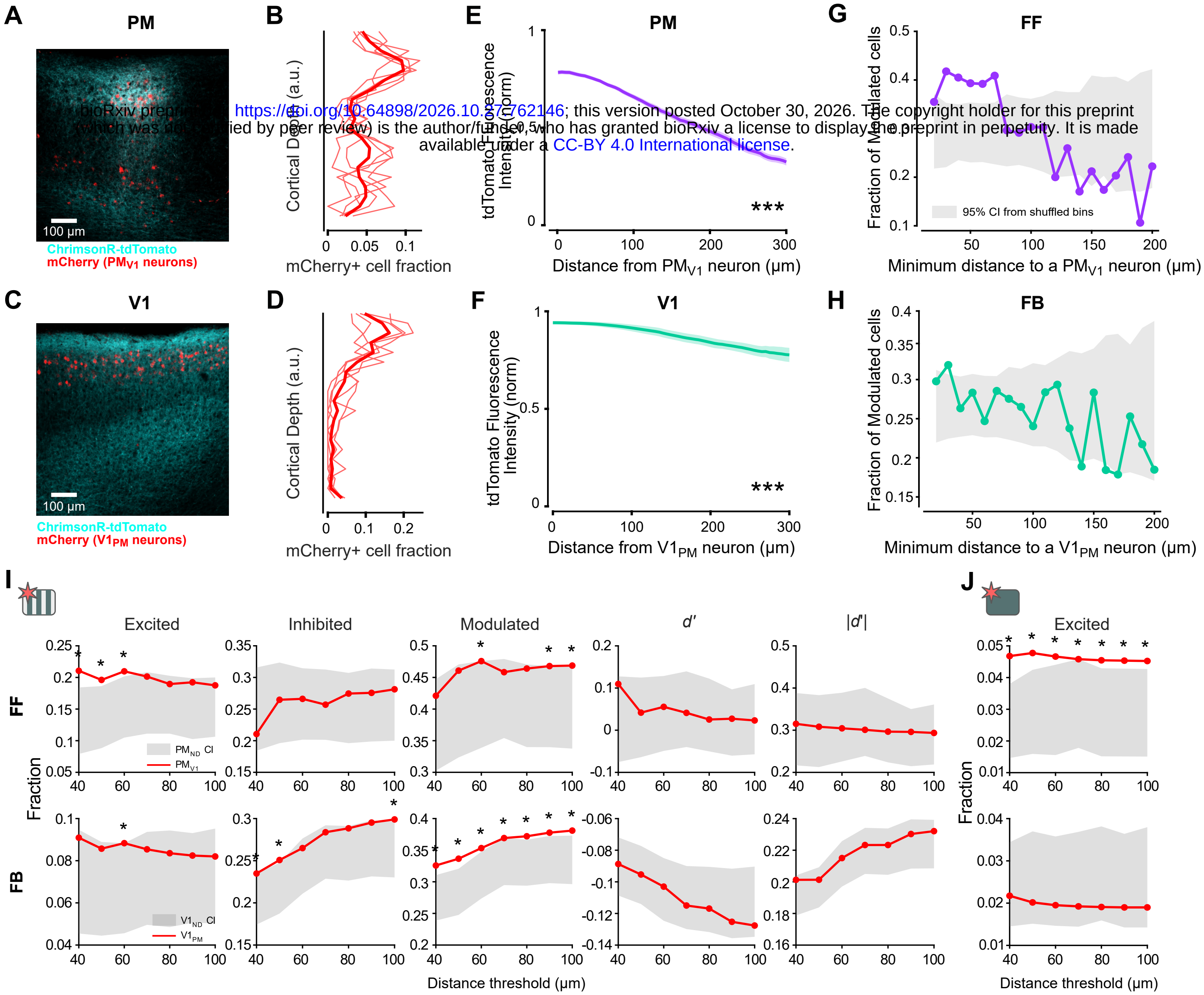

Figure S7: Spatial organisation of the influence of FF and FB photostimulation.

A) Coronal section with $V1_{PM}$ ChrimsonR-tdTomato-expressing axons and PM_{V1} mCherry-expressing retrogradely labelled cells from the FF photostimulation experiments.
B) Laminar distribution of PM_{V1} mCherry-expresing retrogradely labelled cells. Thick line, average, thin line, individual mice (n=5 mice).
C) Coronal section with PM_{V1} ChrimsonR-tdTomato-expressing axons and $V1_{PM}$ mCherry retrogradely labelled cells from the FB photostimulation experiments.
D) Laminar distribution of $V1_{PM}$ mCherry-expressing retrogradely labelled cells (n=5 mice).
E) Fluorescence intensity of tdTomato-expressing FF axons as a function of the distance to mCherry-expressing PM_{V1} cells.Thick line, average; shading, s.e.m across 20 slices from N=5 mice (one-way repeated measures ANOVA, $F_{60,1140} = 210.1$, $p = 3.02 \times 10^{-21}$).
F) Fluorescence intensity of tdTomato-expressing FB axons as a function of the distance to mCherry-expressing $V1_{PM}$ cells (one-way repeated measures ANOVA, $F_{60,480}=21.6$, $p= 9.98 \times 10^{-5}$, n=9 slices, N=4 mice).
G) Fraction of PM neurons modulated by FF photostimulation (excited or inhibited) as a function of their distance to a PM_{V1} neuron. Grey shaded area corresponds to the 95% confidence interval from shuffled bins.
H) Same as G for the fraction PM neurons modulated by FB photostimulation and distance to a $V1_{PM}$ neuron.
I) Same analyses as Fig 6B,C but for different distance thresholds for selecting non-reciprocating neurons in the vicinity of looped neurons. The fraction of excited, inhibited and modulated reciprocating neurons (red line) is compared with shuffled resamples of neighboring non-reciprocating neurons at different distance thresholds (grey distribution, 95% confidence interval from 10.000 resamples). Top row, FF photostimulation. Bottom row, FB photostimulation.
J) Same as I for the fraction of excited cells by photostimulation without visual stimulus.
